# Core competencies for pharmaceutical physicians and drug development scientists

**DOI:** 10.3389/fphar.2013.00105

**Published:** 2013-08-26

**Authors:** Honorio Silva, Peter Stonier, Fritz Buhler, Jean-Paul Deslypere, Domenico Criscuolo, Gerfried Nell, Joao Massud, Stewart Geary, Johanna Schenk, Sandor Kerpel-Fronius, Greg Koski, Norbert Clemens, Ingrid Klingmann, Gustavo Kesselring, Rudolf van Olden, Dominique Dubois

**Affiliations:** IFAPP Working Group on Competencies in Pharmaceutical MedicineNetherlands^†^

**Keywords:** competency-based education, learning outcomes, pharmaceutical medicine, core competencies, pharmaceutical medicine, core competencies, medicines in the 21st century

## Abstract

Professional groups, such as IFAPP (International Federation of Pharmaceutical Physicians and Pharmaceutical Medicine), are expected to produce the defined core competencies to orient the discipline and the academic programs for the development of future competent professionals and to advance the profession. On the other hand, PharmaTrain, an Innovative Medicines Initiative project, has become the largest public-private partnership in biomedicine in the European Continent and aims to provide postgraduate courses that are designed to meet the needs of professionals working in medicines development. A working group was formed within IFAPP including representatives from PharmaTrain, academic institutions and national member associations, with special interest and experience on Quality Improvement through education. The objectives were: to define a set of core competencies for pharmaceutical physicians and drug development scientists, to be summarized in a Statement of Competence and to benchmark and align these identified core competencies with the Learning Outcomes (LO) of the PharmaTrain Base Course. The objectives were successfully achieved. Seven domains and 60 core competencies were identified and aligned accordingly. The effective implementation of training programs using the competencies or the PharmaTrain LO anywhere in the world may transform the drug development process to an efficient and integrated process for better and safer medicines. The PharmaTrain Base Course might provide the cognitive framework to achieve the desired Statement of Competence for Pharmaceutical Physicians and Drug Development Scientists worldwide.

## Introduction

There is a perceived mismatch between the profile of the graduates from academic programs in health care professions and the changing needs of the various health systems around the world. Professional education has not kept pace with these changes, largely because of fragmented, outdated and static curricula that produce ill-equipped graduates. Redesign of professional health education is thus necessary and timely, aiming for transformative learning and interdependency in education. (Institute of Medicine, 2002; Josiah Macy Jr. Foundation, 2008; UK General Medical Council, 2009; Benner et al., 2010; Frenk et al., 2010; The Association of Faculties of Medicine of Canada, 2010).

Transformative learning involves three fundamental shifts: (1) from memorizing facts to search, analysis and synthesis of information for decision-making; (2) from seeking individual professional credentials to achieving core competencies for effective teamwork in health systems, and (3) from non-critical adoption of educational models to creative adaptation of global resources to address local priorities. Outcomes-Based Education or Competency-Based Education has been proposed as a suitable solution for transformative learning. (Dreyfus, 2004; Harden, 2007; Heffron et al., 2007; Gruppen et al., 2010).

*Competency based education* (CBE) is an emerging discourse in the health profession's education and has been adopted by numerous academic institutions and professional associations all over the world, at the undergraduate, postgraduate and continuing professional development (CPD) levels.

The definition of *CBE* is highly variable in the literature. A systematic review of the medical education literature related to CBE definition led to the following proposal: “CBE is an approach to preparing physicians for practice that is fundamentally oriented to graduate outcome abilities and organized around competencies derived from an analysis of societal and patient needs. It de-emphasizes time-based training and promises greater accountability, flexibility and learner centredness” (Frank et al., 2010a). CBE is organized around competencies, or predefined abilities, as outcomes of the curriculum.

*Competency* is defined as “an observable ability of any professional, integrating multiple components such as knowledge, skills, values, and attitudes.” Since competencies are observable, they can be measured and assessed to ensure their acquisition. Competencies can be assembled like building blocks to facilitate progressive development. (Frank et al., 2010b)

Given that in the English language “competency” can be used interchangeably with “competence” (Hager and Gonczi, 1996), in the medical education and assessment literature the term “competency” should be restricted to the skill itself, while “competence” is the ability to perform that skill and the attribute of the performer (Khan and Ramachandran, 2012). Competence is a point on the spectrum of improving performance. A *competent professional* is one possessing the required abilities in all domains in a certain context at a defined stage of education or practice. Competence and performance are different although closely interrelated. Performance can be affected by a number of factors, regardless of competence. (Dreyfus, 2004; Frank et al., 2010a,b).

There is also a growing realization on the concept of “progression of competence” meaning that learners advance along a series of defined milestones on their way to the explicit outcomes goals of training and can perform as per the expectations of the employers and the society at large. (Miller, 1990; Khan and Ramachandran, 2012)

## Competencies in pharmaceutical medicine/drug development sciences

For the past 40 years pharmaceutical medicine has evolved as a medical scientific discipline for the discovery, development, evaluation, registration, monitoring and medical marketing of medicines for the benefits of patients and community health. Pharmaceutical physicians work in industry, drug regulatory authorities and clinical research organizations, but have a close affinity with their medical colleagues in primary and secondary healthcare and at universities.

As a postgraduate medical discipline, pharmaceutical medicine has a recognized international syllabus, training courses with examinations and qualifications, its own research methodologies and embraces new technologies and regulations in pursuit and proof of efficacy, safety and effectiveness of medicines. Organized pharmaceutical medicine is a relatively young medical specialty. Although there are physicians working for pharmaceutical companies worldwide, there is limited awareness of the discipline at the level of the academic and national medical associations, which has contributed to a slow uptake in achieving recognition as a medical specialty. Four countries in Europe: Switzerland, United Kingdom (through the Faculty of Pharmaceutical Medicine), Belgium (through the Belgian College of Pharmaceutical Medicine) and Ireland as well as two in Latin America (Mexico and Argentina) have accepted Pharmaceutical Medicine as a distinct medical specialty. In Europe, pharmaceutical medicine is still in its infancy in most of the countries, and few schools of medicine have a dedicated curriculum.

About 30 national professional members associations from countries all over the world are affiliated to IFAPP (International Federation of Associations of Pharmaceutical Physicians and Pharmaceutical Medicine), a non-profit organization created in 1975 whose main objectives include to foster the development and international recognition of pharmaceutical medicine as a separate medical specialty and to foster the development and training of CME/CPD programs in the discipline. Around 5000 pharmaceutical physicians and other biomedical professionals involved in drug development are part of the global membership. (Stonier et al., 2007).

In spite of the fact that pharmaceutical companies are used to investing heavily in continuing medical education (CME) or CPD for health care providers, very few consistent efforts to fostering education and training among their employees (particularly pharmaceutical physicians) can be cited. This is probably due to the lack of formal certification (other than the professional qualification) required to work as a physician in the pharmaceutical industry. On-the-job experience and training on specific topics have been the traditional approach to individual professional development within pharmaceuticals.

Surveys conducted among 28 IFAPP national member associations showed that only 20% of the membership had received formal postgraduate education in pharmaceutical medicine (Silva et al., 2012). Similarly, surveys conducted among pharmaceutical physicians in the USA showed the respondents lacked formal training in critical areas of drug development (Stonier et al., 2011).

Professional groups, such as IFAPP, are expected to produce the defined core competencies to orient the discipline and the academic programs for the development of future competent professionals and to advance the profession.

PharmaTrain, an Innovative Medicines Initiative project, is a Public Private Partnership of 24 Universities, 13 learned Societies/Associations and several partner training organizations, including Regulatory Authorities, and 15 pharmaceutical companies affiliated to the European Federation of Pharmaceutical Industries and Associations (EFPIA). Another 10 universities from Central Eastern Europe cooperate in the collaborative European Medicines Development Course, CEMDC, coordinated at Semmelweis University in Budapest. In the last 2 years the nucleus of courses sharing the same standards has been set-up with an additional group of 12 universities in all continents.

IFAPP is a founding member of PharmaTrain and has adopted its syllabus (list of topics comprising a subject, discipline or specialty field), and curriculum (guideline to transfer the content of the Syllabus into the modular structure and quality management system). The curriculum also provides the aims and objectives, contents, experiences, outcomes and processes of a program including the methods of learning, teaching, feedback, and supervision.

PharmaTrain has become the largest public-private partnership in biomedicine in the European Continent. PharmaTrain aims to provide courses that are designed to meet the needs of professionals working in medicines development. A modular base Diploma Course, a formal Master's degree and a CPD platform provide the opportunities for education and training in Europe. The quality training program in integrated drug development is now being standardized by a number of academic organizations worldwide. (Klech et al., 2012; PharmaTrain Manual: curriculum, PharmaTrain Manual, 2012).

The PharmaTrain curriculum is based upon *Learning Outcomes* (LO), which are statements of what a student is expected to know, understand and/or be able to demonstrate after completion of a process of learning. LOs are an integral part of the curriculum.

Since most of the current postgraduate programs in Pharmaceutical Medicine worldwide are knowledge-based, there is a need to define a core set of competencies that will be help in the preparation of CBE curricula or to benchmark with the LO of established curricula, such as those from the PharmaTrain Base Course.

The objectives for our work were two-fold: (1) to define a set of core competencies for pharmaceutical physicians and drug development scientists, to be summarized in a *Statement of Competence*; and (2) to benchmark and align these identified core competencies with the LO of the PharmaTrain Base Course.

## Methods

A working group was formed within the IFAPP's Council on Education in Pharmaceutical Medicine (CEPM) including representatives from PharmaTrain, academic institutions and IFAPP's national member associations, with special interest and experience on Quality Improvement through education. The group was also involved in teaching in the discipline at the undergraduate, postgraduate, and CPD levels. Participants were given a presentation defining the scope of the project and relevant definitions. A thorough review and analysis of the core competencies published by academic groups or professional associations related to Pharmaceutical Medicine and clinical research was conducted (Batalden et al., 2002; Frank, 2005; Burke et al., 2008; Calhoun et al., 2008; Joint Royal Colleges of Physicians Training Board, 2009; Koren et al., 2010; Silva, 2010; Weinberger et al., 2010; CTSA, 2011; General Medical Council, 2011; Consortium of Academic Programs in Clinical Research, 2012; Czabanowska et al., 2012; Klech et al., 2012; Leadership Academy NHS UK, 2013). However, this was not intended to be a systematic review, but the collection and evaluation of best practices and recommendations related to competencies. A combination of bibliographic search and individual consultations with related groups was agreed for this exercise. A modified six-sigma approach was used. The domains were identified through benchmarking, alignment and harmonization of domains and competencies from other similar or related groups. The recommendations from the Joint Royal College of Physicians Training Board (Joint Royal Colleges of Physicians Training Board, 2009) were the backbone for this exercise.

The process can be summarized in three phases. In the first (identification) 12 domains and 110 competencies were drafted. After the initial identification of the draft list of domains and their associated competencies, the group members were asked to qualify each competency according to its relevance for inclusion in the model, suggest possible changes in the competency statement, as well as possible changes in the appropriate domain. The work was conducted via teleconferences and face to face meetings during the period September 2011–January 2012.

During the second phase (confirmation) the number of domains and competencies was lowered to 7 and 60 respectively. This second set of proposed domains and competencies and tentative alignment with PharmaTrain LO underwent another process of expert review and discussions within IFAPP-CEPM and PharmaTrain. Individual competencies were edited and reorganized based upon the recommendations. This process was extended till May, 2012 and a draft document was prepared.

In the final phase (validation) the draft document was reviewed by external consultants appointed by both the IFAPP's national member associations and PharmaTrain. The second set of domains and competencies, the statement of competence and the alignment with PharmaTrain LO was then sent for review by and feedback from IFAPP's National Member Associations. All national associations except one accepted the proposed document. The final version was sanctioned at the IFAPP's General Assembly held in Barcelona, Spain on November 17, 2012. The overall initiative was completed within 18 months.

The critical issues considered were: areas and domains for competency, and its intrinsic and extrinsic validity; the descriptors for each competency and their relevance; the level of granularity and comparability with other professions and disciplines, and the level of desired expertise. The group focused only in the cognitive aspects for each proposed competency and conducted a mapping exercise with the LO and Curriculum for the PharmaTrain Base Course. The competencies were verbalized using highest wording associated with the competence category in the revised Bloom's Taxonomy (Bloom, 1956).

## Results

Three areas (Drug Development and Clinical Trials, Regulatory Affairs and Safety of Medicines, Health Care and Professionalism) and 7 core competency domains were identified within the competence framework as follows: Discovery of Medicines and Early Development; Clinical Development and Clinical Trials; Medicines Regulation; Drug Safety Surveillance; Ethics and Subject Protection; Health Care Market Place; Communication and Management. A total of 60 core competencies for Pharmaceutical Physicians and Drug Development Scientists were included within the above mentioned 7 domains (Tables 1–4).

**Table 1:** **Domains and desired competencies for the area of drug development and clinical trials**.

**Domain: discovery of medicines and early development**	**Domain: clinical development and clinical trials**
Evaluates and analyses a disease area within the industry clinical development environment and identifies unmet therapeutic needs	Evaluates the conduct and management of clinical trials within the context of the Clinical Development Plan and working as part of a Team
Evaluates the clinical and non-clinical pharmacology and toxicology evidence for a new candidate for clinical development	Designs and executes confirmatory studies and evaluates the resulting data as applied to the Clinical Development Plan and the TPP
Evaluates and applies the regulatory and ethical aspects underpinning clinical development	Evaluates and interprets the principles for the development of a clinical trial protocol applying principles of GCP in clinical pharmacology
Creates a Clinical Development Plan for a new candidate including a Target Product Profile (TPP)	Summarize the principles of Case Report Form design and clinical data management, including CDISC, EDC, and MedDRA
Designs and executes exploratory studies and evaluates the resulting data as applied to the Clinical Development Plan	Organizes the activities and processes related to the selection and management of sites for individual or multi-center clinical trials
Contrast the advances made in the clinical pharmacology of a new medicine in a stepwise manner with the overall Clinical Development Plan and the TPP	Supports and provides the clinical input into the design and review of a Statistical Analysis Plan
Defends the statistical principles for the design, conduct and assessment of exploratory studies	Appraises and reviews relevant literature and other sources and writes manuscripts for publication
Justifies the various end-points used in the clinical development program	
Appraises suspected adverse reactions during Exploratory development	Interprets and explains the outcomes of clinical studies

IFAPP/PharmaTrain 2012.

**Table 2:** **Domains and desired competencies for the area of regulatory affairs and safety of medicine**.

**Domain: medicines regulation**	**Domain: drug safety surveillance**
Summarizes the legislative framework supporting the development and registration of medicines, ensuring their safety, efficacy and quality	Contrasts the key regulatory requirements for pharmacovigilance, both in the major ICH regions and locally, and their historical background
Describes the regulations related to post-authorization safety monitoring and reporting procedures	Organizes the medical assessments required to meet the requirements for drug safety reporting both at the level of the individual patient (case report) and aggregate report
Justifies the significance of regular product Safety Update Reports to the regulatory agencies and participates in their preparation and review	Summarizes the spontaneous reporting and signal detection methodologies and assesses medically Adverse Event/Adverse Drug Reaction reports as part of causality assessment
Evaluates the unlicensed use of medicines and ensures patient safety is paramount	Summarizes the principles and methods of evaluation of risk and benefit balance and the principles and methods for managing risk to patient and clinical trial subjects
Describes procedures in the development and renewal of Marketing Authorizations	Discriminates the variety of regulatory actions possible to address concerns about patient safety
Designs, prepares, reviews and evaluates Clinical Overviews for regulatory submission	Describes the importance of communication of safety issues, the variety of formats required to meet audience needs and contributes to the development of such communications
Describes the legal framework for clinical trials and the requirements in different regions and perceived problems associated with global drug development	Evaluates a safety issue and establish a crisis management team, recognizing the key functional areas to be represented and their roles and responsibilities
Describes the mechanisms for wider availability of medicines, and undertakes or contributes to product deregulation	Appraises the areas of progress, likely major advances and future challenges in drug safety and pharmacovigilance
Organizes the investigation of product defects, counterfeit products and other miscellaneous pharmaceutical procedures and requirements	
Describes the principles and process of regulation of medical devices and biotechnology formulations	

IFAPP/PharmaTrain 2012.

**Table 3:** **Desired competencies in communication and management (health care and professionalism area)**.

**Domain: communication and management**
Describes the principles and practices of people management and leadership to apply them within their own working environment; sets learning and improvement goals
Ensures that the knowledge, skills and behaviors associated with the competent practice of pharmaceutical medicine are communicated effectively, using the best techniques and practices whilst participating in the education of colleagues and stakeholders
Organizes networks and builds and maintains relationships, encouraging contribution and working with interprofessional teams to meet the business objectives
Supports the success of the organization by actively contributing to develop strategic plans to achieve goals, manage resources and people, and leverage performance
Ensures organizational excellence by developing critical evaluation skills, encouraging improvement and innovation in managing change
Identifies strengths, deficiencies and limits in one's knowledge and expertise
Works effectively as a member or leader of a healthcare team or other professional groups
Explains his/her accountability to key stakeholders, society and the profession of pharmaceutical medicine
Applies quality and performance improvement concepts to address organizational performance issues

IFAPP/PharmaTrain 2012.

**Table 4:** **Domains and desired competencies in the area of health care and professionalism**.

**Domain: health care market place**	**Domain: ethics and subject protection**
Describes the commercial healthcare environment in which pharmaceutical medicine operates, identifying the contribution of laws and of regulators and other stakeholders in the decision making for prescribing medicines	• Evaluates the impact of cultural diversity and the need for cultural competency in the conduct of clinical trials and other business activities
Summarizes the key elements involved in medical/marketing communication in the healthcare environment and explains the importance of compliance with regulation in this context	• Describes the ethical and professional issues (conflicts of interest, plagiarism, authorship and intellectual property) associated with clinical research, drug development and commercialization on the production of scientific knowledge
Describes the pharmaceutical industry: internal environment, structure and function, key stakeholders and commercial drivers and explains how these business elements impact on the broader healthcare market place	• Describes the significance of historical abuses on the evolution of principles of human subject protection
Describes the information required to undertake a commercial analysis of the market potential for a pharmaceutical product/candidate within the industry business environment	• Evaluates the key documents related to the ethical conduct of clinical trials and pharmaceutical marketing operations
Appraises the commercial competitor environment when evaluating the opportunity for new medicine under development or a currently marketed product	• Describes the ethical issues involved when dealing with vulnerable populations and the need for additional safeguards
Describes the interface between pharmaceuticals and the external stakeholder environment and the challenges balancing the commercial and professional aspects in making ethical judgments within the legal/regulatory framework	• Compares the requirements for human subject protection and privacy under different national and international regulations
	• Summarizes the principles of Corporate Social Responsibility

IFAPP/PharmaTrain 2012.

**Figure 1:**
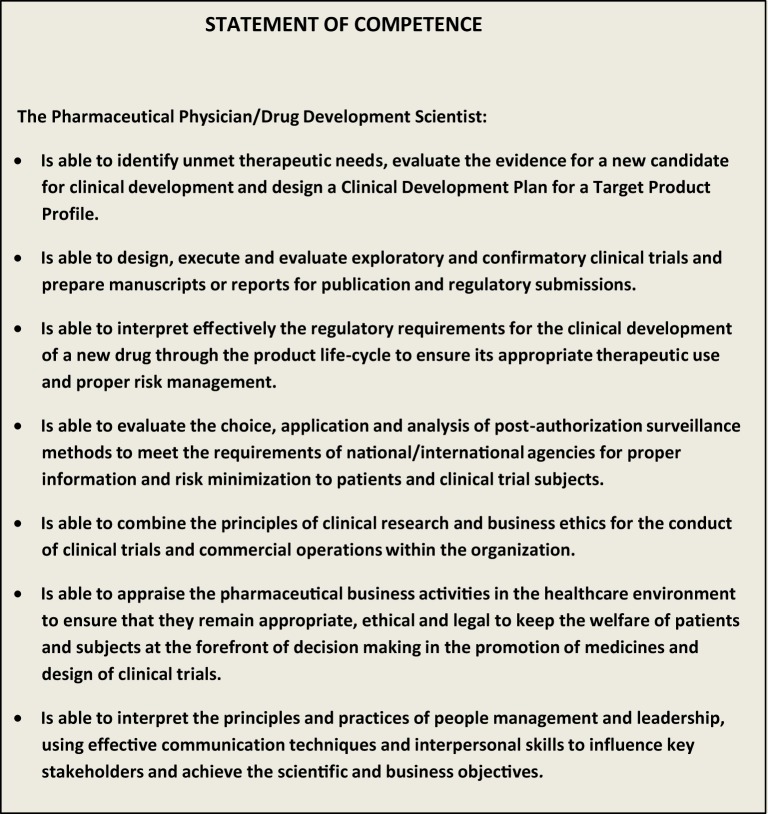
**Statement of competence in pharmaceutical medicine**. IFAPP/PharmaTrain 2012.

A Statement of Competence summarizing the competency domains was prepared (Figure 1). As discussed above, this is a succinct description for a competent professional able to successfully participate in any stage of the product life-cycle management.

The LO of the PharmaTrain Base Course were successfully aligned (93%) with the above mentioned competencies.

## Discussion

The competencies are intended to serve as a resource and guide for those interested in improving the quality and accountability of pharmaceutical medicine education and training. They were developed with respect for the uniqueness and diversity in the complex world of medicines development. Therefore, the model may foster further granularity and thus identifying specific sub-competencies and specialty competencies that apply to specific functions in clinical research and drug development.

On the other hand, the competencies are anticipated to serve as a useful guide for education providers (academic or not) to adapt the related contents in their existing courses so that potential students can effectively update their understanding and skills sets in medicines development. The primary vision for this competency model is the availability of professionals more fully prepared for the many challenges and opportunities in pharmaceutical medicine in the next decade.

Competency-based profiles of key jobs in medicines development can be effectively prepared. Standardized job descriptions for various functions could be developed globally. The effective implementation of training programs using the competencies or the PharmaTrain LO anywhere in the world may transform the drug development process to an efficient and integrated process and the product lifecycle management would turn into the availability of better and safer medicines. Additionally, it would provide reassurance to stakeholders of the drug development process that it is in the hands of competent people who are measured against a set of performance standards.

Whilst the process model we used to define the core competencies was inclusive, involving all governance bodies within IFAPP and PharmaTrain as well as key stakeholders within an acceptable timeframe, only the cognitive aspects were included. Further work is needed to define the skills and behaviors involved in each competency. Competency model development are iterative processes, and our model will have to be regularly updated as the competencies are deployed and used for professional, academic or self-assessment purposes. Continued dialogue regarding the use of the competencies, their relevancy, and ongoing changes in the fields of pharmaceutical medicine and other drug development sciences will make the changes imperative. Competency sets generally have a life span of 3–5 years (Batalden et al., 2002; Calhoun et al., 2008) and it will be soon time to revisit the set and initiate new activities for further refinement and updating in line with new thinking and future challenges in the field. The model cannot remain static. PharmaTrain is rolling out a pilot experience in establishing the PharmaTrain Specialist in Medicines Development (www.pharmatrain.eu) based upon the core professional competencies.

Other professional groups involved in clinical research are working to define the roles and competencies of individuals who work in specific content areas, including physician investigators, nurses, investigational site staff as well as other professions involved in regulatory affairs, project management, translational science and comparative effectiveness (CTSA, 2011; Jones et al., 2012). Each group is aiming at the same target: better education of the future workforce of scientists and clinical research personnel. The identification and alignment of competencies among the various groups would give opportunities for inter professional education as well as the identification of new levels of competence and would help in defining a career path for pharmaceutical health professionals.

The general agreement and implementation of core competencies is essential to the ultimate evolution of accrediting bodies which will define the standardization of accredited programs for postgraduate and CPD programs and insure their quality. This will also result in a definition of what we expect from an entry level, a mid-level or advanced level professional in drug development sciences and will certainly help in the related professional certification. PharmaTrain and IFAPP should define joint initiatives to make this happen. Further consultations and proper feedback from the IFAPP affiliated national member associations as well as from key experts in the field would be pursued.

In conclusion: A basic set of core competencies and Statement of Competence are now available for use by IFAPP and serve as guidance to National Member Associations and individuals involved in pharmaceutical medicine and medicines development. The PharmaTrain Diploma Base Course might provide the cognitive framework to achieve the desired Statement of Competence for Pharmaceutical Physicians and Drug Development Scientists worldwide and this can be extended by on the job mentorised CBE for awarding the Specialist in Medicines Development.

### Conflict of interest statement

The authors declare that the research was conducted in the absence of any commercial or financial relationships that could be construed as a potential conflict of interest.
