# Corrigendum: Core competencies for pharmaceutical physicians and drug development scientists

**DOI:** 10.3389/fphar.2014.00297

**Published:** 2015-01-15

**Authors:** Honorio Silva, Peter Stonier, Fritz Buhler, Jean P. Deslypere, Domenico Criscuolo, Gerfried Nell, Joao Massud, Stewart Geary, Johanna Schenk, Sandor Kerpel-Fronius, Greg Koski, Norbert Clemens, Ingrid Klingmann, Gustavo Kesselring, Rudolf van Olden, Dominique Dubois

**Affiliations:** International Federation of Associations of Pharmaceutical Physicians and Pharmaceutical Medicine, Special Secretariat ServiceWoerden, Netherlands

**Keywords:** pharmaceutical medicine, medicines development, drug development scientists, pharmaceutical physicians, corrigendum, competency based education, learning outcomes, core competencies

The total number of competencies mentioned in the Abstract and pages 4 and 5 of the paper are erroneously reported as 60. The actual number is 57. Apologies for the involuntary mistake and confusion created.

## Conflict of interest statement

The authors declare that the research was conducted in the absence of any commercial or financial relationships that could be construed as a potential conflict of interest.

